# Enslaving Minds: On Freedom of Thought and the Exploitation of Mental Vulnerabilities

**DOI:** 10.1007/s12152-025-09620-6

**Published:** 2025-11-08

**Authors:** Sjors Ligthart

**Affiliations:** 1 Tilburg University, Tilburg, the Netherlands; 2 Utrecht University, Utrecht, the Netherlands

**Keywords:** Freedom of thought, Absolute protection, Exploitation, Manipulation, Informed consent, Human rights

## Abstract

One central principle often derived from the right to freedom of thought (RFoT) is that persons’ inner thoughts shall not be impermissibly altered. Since a clear definition of ‘impermissible alteration’ of thought is lacking, the meaning and scope of this principle are largely uncertain. Scholars are now exploring how to operationalise the notion of ‘impermissible alteration’ of thought. For this, some have appealed to the concept of ‘manipulation’, proposing that mind interventions plausibly infringe the RFoT if they are manipulative. This paper argues that the appeal to manipulation is unpersuasive. It explores the potential of the distinct notion of exploitation, which is, unlike manipulation, an international legal concept that underpins absolute prohibitions in human rights law.

## Introduction

Since recent years, scholarly interest in the human right to freedom of thought (RFoT) has been growing [1–8]. This is mainly a response to the emergence of modern technologies that increasingly enable others to interfere with people’s inner mental states. Examples include practices that aim to *influence* what other people think, feel, or decide, such as through microtargeting, hypernudging, dark patterns, and neurotechnologies that stimulate the brain. According to scholars and human rights bodies, the RFoT holds great promise for providing robust legal protection to the human mind in the twenty-first century [e.g., 2–9, 48]. Therefore, scholars are now investigating how to interpret, develop and, possibly, how to reconstruct the RFoT, so as to ensure practical and effective human rights protection against unsolicited interferences with our brains and minds.

One central principle often derived from the RFoT, is that persons’ inner thoughts shall not be *impermissibly altered* [9, 10]. At first sight, this principle seems highly relevant to the (im)permissibility of mind interventions^1^ that aim to change what other people think. However, since a clear definition of ‘impermissible alteration’ is lacking, the meaning and scope of this principle are largely uncertain – and so are its implications for mind interventions [2]. For example, the former Special Rapporteur on Freedom of Religion or Belief, Ahmed Shaheed, has indicated that whereas coercing people to change their thoughts is plausibly prohibited, the use of rational persuasion is normally not [9]. The area in between is, however, grey. For example, what about altering a person’s thoughts, feelings or intentions through traditional advertising, nudging, online marketing, subliminal stimuli, dark patterns, or AI-driven persuasion strategies? Established principles to determine whether such mind interventions may violate the RFoT are lacking. As a consequence, Istace argues, the right not to have one’s thoughts impermissibly altered remains “highly opaque” [2, p. 15]. In a recent report, the Advisory Committee of the UN Human Rights Council highlighted the need to determine what constitutes an ‘impermissible interference’ with freedom of thought [11, fn 18].

To clarify the meaning, scope and potential implications of the RFoT vis-à-vis (traditional and modern) mental influences, scholars are now exploring how to operationalise the notion of ‘impermissible alteration’ of thought. For example, some have appealed to the concept of ‘manipulation’, proposing that a mind intervention plausibly infringes the RFoT if it is *manipulative* [3, 7, 12]. As the former Special Rapporteur on Freedom of Religion or Belief observed: a “growing body of legal scholarship supports the claim that freedom of thought includes freedom from manipulation” [9, par. 35].

The present paper, however, challenges the persuasiveness of the appeal to manipulation in this context. Instead of considering manipulation, this paper aims to explore the normative relevance of a distinct concept, that is: *exploitation*. The reason is that, first, manipulation is an ambiguous and unspecified notion in and of itself. For example, Susser, Roessler and Nissenbaum consider manipulation a “tricky term”, which, they argue, only requires one necessary condition: that the influence on another person’s decision-making is *hidden* or *covert* [13, p. 26–27]. Many such hidden mental influences exist in a variety of contexts; not all of them will plausibly infringe the RFoT. As Sunstein writes: “the idea of a legal right not to be manipulated, to be recognized and enforced by regulators, runs into serious challenges, in part because of definitional problems, which include the risk of overbreadth” [14, p. 1960].

Second, manipulation is a notion that human rights law is not typically familiar with. Exploitation, by contrast, is an international legal concept that is frequently appealed to in the interpretation and adjudication of international law. For example, in both human rights law and international criminal law, the notion of exploitation is central to legal prohibitions of slavery, servitude and “lesser types of human exploitation” [15–17]. Furthermore, exploitation is also a central notion of some theories on the criminalization of harmless conduct [18].

Third, since the RFoT is usually conceived of as having an *absolute* dimension,^2^ clarifying the meaning and scope of ‘impermissible alteration’ of thought faces a very specific constraint. The challenge is not only to clarify why certain forms of mind interventions are *prima facie* impermissible under the RFoT, but rather why those practices should be prohibited in *absolute* terms – without any exception whatsoever. In philosophy, manipulation is generally conceived of as ‘just’ a *pro tanto* and/or *prima facie* wrong, which can sometimes be justified [19, par. 3.1]. However, protecting against such justifiable wrongs does not fit within an absolute understanding of the RFoT, not allowing for any justifications whatsoever.^3^ Interestingly, in human rights law, the ‘exploitation’ of persons and their bodies has been considered the central moral wrong underpinning the absolute prohibition of slavery and lesser types of human exploitation, such as servitude and human trafficking [15, 17].^4^ Perhaps, the exploitation of people’s minds may likewise produce a moral wrong that could justify the absolute prohibition of a specific set of mind interventions by the RFoT.

The aim of this paper is thus to explore the potential relevance of the notion of ‘exploitation’ to elucidate the meaning of the RFoT, by giving substance to “impermissible alteration” of thought. Before proceeding, let me clarify a few points. First, in accordance with the dominant view in human rights scholarship, I assume that the RFoT has an absolute dimension, meaning that infringements of (some elements of) the right can never be justified; no balancing of interests.^5^ Second, the aim of this paper is not to draw a perfect analogy between (modern forms of) slavery and (emerging) mind interventions. Rather, acknowledging the many differences between them, the aim is simply to explore whether the conceptual justification underlying the absolute prohibition of slavery and lesser forms of human exploitation, could add to our understanding and the operationalisation of the RFoT’s absolute dimension. Third, when speaking of the RFoT, I refer to the right as guaranteed by Article 18 ICCPR and Article 9 ECtHR. Meanwhile, some parts of the analysis may equally apply to the RFoT as guaranteed in other human rights instruments.

The paper will proceed as follows. First, a brief overview is provided of recent proposals to operationalise the ‘impermissible alteration’ of thought. Second, the concept of exploitation will be considered from the perspective of international law, followed by, third, an exploration of whether the ‘exploitation’ of people’s minds could be a relevant consideration to defining the boundaries of the RFoT’s absolute dimension, by clarifying the meaning and scope of the freedom from ‘impermissible alteration’ of thought.

## Operationalising ‘Impermissible Alteration’ of Thought: Manipulation and Mental Autonomy

In 2017, human rights lawyer Susie Alegre raised awareness of digital technologies that threaten freedom of thought. In the twenty-first century, she wrote, “it is increasingly clear that there are many ways in which our minds can be manipulated. Marketing, advertising, governmental and political organisations are all exploring the most effective ways to change the way we think” [20, p. 227]. Assuming that the RFoT encompasses a “right not to have one’s thoughts or opinions manipulated”, Alegre highlights the fundamental question of where we should draw the line between legitimate persuasion and unlawful manipulation [20, p. 227]. While setting aside the drawing of this line for further investigation, Alegre suggested that “any technology designed to manipulate the way we think or feel would not be permissible” [20, p. 228]. This, she predicts, will have significant implications for marketers, advertisers and the governmental use of nudge technology, all aiming to influence mental states and, ultimately, the behaviour of users and citizens.

Along the same lines, considering modern means of influencing people’s thoughts and emotions, psychologist Simon McCarthy-Jones has stressed the need to operationalise the RFoT. Like Alegre, McCarthy-Jones assumes that the RFoT includes a right “not to have one’s thoughts manipulated” and calls for drawing a line “between legitimate influence and illegitimate manipulation” [7, p. 11]. A key problem that he observes is the lack of a clear definition of ‘manipulation’, which complicates the operationalization of *illegitimate* manipulation. To counter this, McCarthy-Jones proposes to understand the RFoT as primarily protecting our interest in mental autonomy; any endeavour to operationalise the RFoT should begin by identifying the mental processes that enable mental autonomy, such as attentional and cognitive agency. Circumventing such processes could be defined as the manipulation of thought, some of which may be illegitimate [7].

Keese and Leiser likewise appeal to the idea of protecting mental autonomy as “a guiding interpretative tool” to identify online manipulative practices that may infringe the RFoT [12]. Although it has been argued that manipulation generally undermines autonomy, Keese and Leiser argue that, given the RFoT’s absolute nature, only a narrower set of manipulative practices would plausibly infringe the right: “those in which mental autonomy is threatened or undermined in a sufficiently ‘global’ manner” [12, p. 329]. They suggest the relevance of four factors to assess whether online manipulation infringes the RFoT: (1) its concealment or obfuscation; (2) the vulnerability of the victim together with power and information asymmetry; (3) whether the manipulative practice aims to modify thought that attains a certain level of cogency, seriousness, cohesion and importance and/or targets mental processes intimately linked with core aspects of autonomy in the forum internum; and (4) the frequency and duration of the practice.^6^

More examples can be given of recent proposals to identify specific forms of manipulation that plausibly infringe the RFoT [3], but these suffice to illustrate how the argument goes and where the difficulties lie. Generally, the argument starts from the assumption that the RFoT includes a right against having one’s thoughts ‘manipulated’. From there, the main challenge is to define manipulation and identify when, which and why specific forms of manipulation are illegitimate and should therefore be prohibited by the absolute RFoT. To address this issue, mental autonomy is invoked as the central interest protected by the RFoT. Manipulative practices that undermine mental autonomy to a certain (sufficient) degree will qualify as impermissible manipulation and therefore infringe the absolute RFoT.

One issue that remains unclear, however, is why the authors limit their analyses to the *manipulation* of thought in the first place. Usually, human rights commentators do not employ the specific language of ‘manipulation’ when considering the substantive freedoms implicit in the RFoT. Rather, they refer to the freedom from impermissible *influence* or *interference* with inner thought [21, p. 503],^7^ stressing that States may never *interfere* with the most intimate and inner sphere of persons, for instance, by employing coercion to *change* personal thoughts [10, p. 738].^8^ Authors like Alegre and McCarthy-Jones cite the work of these commentators, but then they seem to switch language and introduce the notion of *manipulation* instead [7, 10, 20, p. 225; ]. Meanwhile, it remains unclear on what precedents, theory, or arguments the authors base themselves when claiming that the RFoT implies a right not to have one’s thoughts manipulated. Perhaps, the implicit assumption is that manipulation undermines (mental) autonomy,^9^ which is, according to some, the central personal interest protected by the RFoT [2]. Or perhaps there is another reason why these authors favour reference to a right against the manipulation of thought, rather than to the freedom from impermissible interference with, or alteration of, thought. In the absence of clear arguments, we are left to speculate.

However, confining the freedom from ‘impermissible alteration’ of thought to a right not to have one’s thoughts ‘manipulated’, raises a variety of questions and challenges. First, as highlighted in the introduction, the RFoT is usually considered to be an absolute right,^10^ while manipulation is generally conceived of as a *pro tanto* and/or *prima facie* wrong, which can sometimes be justified [19]. Providing absolute protection against justifiable wrongs is, however, conceptually untenable. Furthermore, it is unclear which influences qualify as manipulation, and for what reasons.^11^ Moreover, whereas some forms of manipulation may well infringe the RFoT, for instance, because they subvert mental autonomy, the scope of the RFoT is plausibly broader: it also applies to practices that undermine or bypass mental autonomy without being manipulative. For example, next to manipulation, the Special Rapporteur has suggested that using *coercion* to make a person change their thoughts will usually qualify as the impermissible alteration of thought, as will the direct *modification* of thoughts by altering brain chemistry. Although not manipulative, both coercion and direct brain modification disrespect the person’s mental autonomy, which can serve as a reason to qualify them as the impermissible alteration of thought as is prohibited by the RFoT.

If, for the applicability of the RFoT, the central question is ultimately whether a mind intervention delimits or undermines a person’s *mental autonomy* (as suggested by McCarthy-Jones and Keese and Leiser), then it may be more straightforward to develop a criterion that likewise centres on the person’s mental autonomy – rather than on one specific way of undermining it, like through manipulation. For example, Istace recently suggested a guiding normative framework for the moral evaluation of “undue mind interventions”, which he argues should inform the interpretation of the legal RFoT insofar it protects against the impermissible alteration of thought [2]. He proposes that mind interventions are morally undue when an agent “intentionally undermines a subject’s mental autonomy by bypassing, distorting, or disarming the mental abilities that enable it” [2, p. 6]. This may include some forms of manipulation, as well any other non-manipulative intervention that bypass, distort or disarm the mental capacities necessary for mental autonomy.

However, as Istace rightly points out, while it is one thing to identify *prima facie* impermissible mind interventions, it is yet another thing to determine which types of interventions should be *absolutely* prohibited within the unconditional dimension of the RFoT. Some ways of influencing people’s minds may be legitimate and justifiable in some cases, such as governmental nudging to steer individuals towards getting themselves vaccinated for public health reasons [22], or the nonconsensual administration of antipsychotics to a psychiatric patient in order to prevent imminent harm [4, 23]. Hence, according to Istace, effective and comprehensive protection against mind interventions in human rights law should plausibly have both (1) a relative dimension (where mind interventions can sometimes be justified), complemented by (2) an absolute dimension (where a limited number of interventions are prohibited without any exception) [2].

This idea of a multi-layered legal framework that protects against unsolicited mind interventions finds support in the literature and in the established framework of human rights law [4, 12]. For example, Bublitz has shown that the *relative* human right to bodily and mental integrity forms a first comprehensive layer of protection against unsolicited mind interventions, which can sometimes be outweighed by countervailing factors, such as the rights and interests of others [24]. This relative protection is complemented by a second, impenetrable layer of human rights protection, which includes the absolute dimension of the RFoT [24]. As Keese and Leiser argue, given the absolute dimension of the RFoT, “it is neither tenable nor desirable to view *every type* of manipulative practice as a violation of this right” [12, p. 329, emphasis added]. Rather, a broad range of mental influences should plausibly be considered to infringe the relative right to respect for private life, which includes a right to physical and mental integrity, while only a narrower subset of sufficiently severe interventions would violate the absolute RFoT [12]. In other words: relative protection is the central norm, absolute protection the exception.

From here, a crucial yet unanswered question arises: how should we determine what types of mind interventions are typically covered by either the relative or absolute dimension of human rights protection of the mind? How should judges assess whether a mind intervention either infringes the relative right to mental integrity or, rather, violates the absolute dimension of the RFoT? [25] In other words: what types of mind interventions should qualify as the ‘impermissible alteration’ of thought in the meaning of the RFoT?

Istace has suggested that undue mind interventions that “profoundly impact an individual’s fundamental interests” may justify absolute protection by the RFoT. As candidate examples, he mentions impermissible interferences with political opinions or sexual orientation [2].^12^ This seems plausible. For example, according to the European Court of Human Rights, the absolute RFoT only applies to “those views that attain a certain level of cogency, seriousness, cohesion and importance.”^13^

Apart from the profoundness of the effects a mind intervention may have on the *victim’s* personal interests, it is worth exploring the potential relevance of whether the acts by the *performer* may qualify as being ‘exploitative’.^14^ The next section discusses the concept of exploitation in international law, followed by an exploration of its potential relevance for the operationalising of the RFoT.

## Exploitation in International Law: Serious Wrongs, Absolute Prohibitions

In international law, the concept of exploitation manifests itself, generally, in two different ways: on the one hand, as the exploitation of *things,* such as natural resources like iron, gas and oil, and on the other hand, as the exploitation of *human beings* [26,2, p. 21]. Whereas the former is usually conceived of as morally neutral, the latter has an inherent “evil connotation”, always being “pejorative”, so it was agreed upon during the negotiations of the UN Supplementary Convention on the Abolition of Slavery [27, p. 311, 316–317]. In the same vein, Joel Feinberg notes that the exploitation of persons most frequently tends to be pejorative; it refers to using someone for one’s own ends, “which is somehow wrongful or blameworthy, whether it wrongs the other person or not” [18, p. 177]. The exploitation of persons is the central notion that binds together a variety of practices that are strictly prohibited within international law, including slavery, servitude, human trafficking and compulsory labour [15, 17, 26, p. 346–347; ]. But what does it mean to exploit a human being? And how does the law protect against different forms of human exploitation?

At the most basic level, to exploit someone means to *use* them [18, 28, 29]. However, as Allen Wood explains, exploitation differs from mere use “in that what is exploited is used as part of a plan by the exploiter to achieve some end, in which the exploiter exercises some kind of *domination* or *control* over what is exploited” [28, p 43, emphasis added]. Furthermore, most philosophers agree that exploitation involves the taking of *unfair advantage* over another person, and that, in order to exploit someone, the exploiter must benefit at the expense of the exploited [30, par. 2].^15^ Often, reference is made to using or abusing a person’s *vulnerability*. For example, according to Wood, there are two central elements in the exploitation of human beings: “(a) the vulnerability of the exploited of which the exploiter makes use in gaining control; and (b) the capacity or other benefit of which the exploiter makes use through exercising this control over the exploited” [28, p. 43].

As Feinberg explains, virtually every capacity or trait is in principle exploitable, including “occurrent mental states of relatively brief duration (…) such as particular states of joy or grief, anger or love” [18, p.181]. Meanwhile, when considering human-to-human exploitation, we usually do not speak about certain ‘capacities’ or ‘traits’ as the object of exploitation. Rather, we say that the *person themselves* is exploited. We do this, Wood supposes, if we consider the control of the exploiter over the exploitee to be “sufficiently global”, or if the object of exploitation is “intimately enough involved with the very personhood of the exploited”, so that we consider the entire person to be controlled and made use of [28, p. 43].^16^

The relevant elements of domination and control are also central to the understanding of the severest form of human exploitation in international law, i.e., slavery. As Jean Allain writes: “where a person controls another such as they would control a thing they own, this will be deemed, in law, slavery” [15, p. 5]. Such control tantamount to possession significantly restricts the person’s autonomy and destroys their agency. It deprives the person from making decisions themselves to the extent that their exploitation can be completed, by using, selling, or managing them [15]. For example, according to Article 1(1) of the UN Slavery Convention, “Slavery is the status or condition of a person over whom any or all of the powers attaching to the right of ownership are exercised.” As guideline 2 of the Bellagio–Harvard Guidelines on the Legal Parameters of Slavery explain:

In cases of slavery, the exercise of “the powers attaching to the right of ownership” should be understood as constituting *control* over a person in such a way as to significantly deprive that person of his or her individual liberty, with the intent of exploitation through the use, management, profit, transfer or disposal of that person. Usually this exercise will be supported by and obtained through means such as violent force, deception and/or coercion. [emphasis added]

In human rights law, slavery and servitude are *inter alia* prohibited by Articles 8 ICCPR and 4 ECHR. These prohibitions are absolute and non-derogable. No exceptions whatsoever [31,32, p. 286]. In the context of international criminal law – where enslavement constitutes a crime against humanity – the ICC Office of the Prosecutor has stressed that “Slavery and the slave trade are foundational international crimes. International law accords them the utmost importance, that of peremptory norms from which no derogation is permitted, designed to prevent violations of liberty, dignity, autonomy and humanity” [33, p. 4]., p. 220; 

Apart from slavery, international law also protects against “lesser types of human exploitation” [15, p. 1].^17^ A general definition of such types is missing, but different (non-exhaustive) examples can be found in the law. For example, the notion of exploitation is central to the prohibition of human trafficking [17]. As Sellers and Kestenbaum explain, the purpose of criminalizing human trafficking is to prevent individuals from being subjugated to forms of exploitation [34, p. 19]. Article 3 of the Palermo Protocol defines human trafficking as the “recruitment, transportation, transfer, harbouring or receipt of persons (…) for the purpose of exploitation.”^18^ Exploitation in the meaning of this provision includes, but is not limited to, “the exploitation of the prostitution of others or other forms of sexual exploitation, forced labour or services, slavery or practices similar to slavery, servitude or the removal of organs.” According to the UN Office on Drug and Crime, the meaning of exploitation in this context is broadly consistent with the general definition of one person taking unfair advantage of another person, their vulnerability or their situation [35, p. 39]. It has been suggested that human trafficking is, like slavery and servitude, prohibited in an absolute and non-derogable way, but this is open to discussion.^19^

The legal provisions concerning slavery and human trafficking provide some descriptive examples of how the concept of exploitation manifests in international law. Meanwhile, they do not clarify much about the *moral weight* and *moral force* of exploitation as the central concept underlying these (nearly) absolute prohibitions of (modern) slavery. Whereas the ‘moral weight’ of exploitation refers to the intensity of its wrongness, ‘moral force’ refers to the moral reasons for action that exploitation may call for, including whether exploitative actions should be prohibited by law [30, p. 28, 279–280]. As Marija Jovanovic rightly points out, the question of moral force is significant in order to determine the rationale and scope of human rights that prohibit human exploitation, such as the absolute prohibition of slavery, servitude and the prohibition of forced compulsory labour [17, p. 691].

In philosophy, different positions have been defended regarding the wrongness of human exploitation, and different conclusions have been drawn from those positions with regards to how society should deal with it. For example, exploitation can be considered wrong because it is *harmful*. As Zwolinski, Ferguson and Wertheimer explain, “[w]hen exploitation is harmful and nonconsensual, issues of both moral weight and force are relatively unproblematic. Whatever the added moral importance of the gain to *A* from the harm to *B*, it is certainly at least *prima facie* wrong for *A* to harm *B* and it seems that the state is at least *prima facie* justified in prohibiting or refusing to enforce such transactions” [30, par. 3]. In the same vein, Feinberg argues that when *A*’s conduct exploits *B* and adversely affects their interest without valid consent, the harm principle can validate prohibitions of such wrongful harm-doing [18]. According to Wertheimer, exploitative actions may, apart from the exploitee, also harm others, including society at large, which may justify prohibitions of such exploitative actions [36, p. 300–309].

Next to harm, it has been argued that the wrongness of exploitation sometimes lies in the *unfair* treatment of the exploited party [18, p. 199]. In cases where exploitation is harmless (or mutually advantageous and consented to [36]), the exploitative action can still be considered a *prima facie* wrong because it is unfair [18, 36]. According to Feinberg, unfair exploitation can, however, be “justified on balance”, depending on all circumstances of the case.^20^

Apart from harm and unfairness, some theorists have argued that what really explains and justifies our moral belief that human exploitation is wrong, is because it “is *degrading* to have your weaknesses taken advantage of, and *dishonorable* to use the weaknesses of others for your own ends” [37, p. 151, emphasis added]. According to Wood, “it is an affront to people’s *human dignity* to have their weaknesses used, and shameful to use the weaknesses of others” [37, p. 158, emphasis added]. Elsewhere, Wood writes that exploitation is “degrading to the exploited because it deprives them from freedom, which is fundamental to their human dignity” [28, p. 46]. In the same vein, Jonathan Wolff turns to Kant’s Categorical Imperative to account for the wrongness of human exploitation: “To exploit someone is to treat a person purely as a means to your own ends, not as an ‘end in themselves’” [29, p. 112], which disrespects human dignity.

In international law, the need to respect human dignity (and, accordingly, to treat persons as ends in themselves, not only as means) appears a central reason underlying strict and absolute prohibitions of human exploitation [15, 17, 26].^21^ For example, from the drafting history of the Universal Declaration of Human Rights, it becomes clear that slavery and compulsory labour had been considered “inconsistent with the dignity of man and therefore prohibited”.^22^ The link between respecting human dignity and prohibiting human exploitation is also apparent in Article 5 of the African Charter on Human and Peoples' Rights, which guarantees to everyone a “right to the respect of the dignity inherent in a human being and to the recognition of his legal status. All forms of exploitation and degradation of man, particularly slavery, slave trade, torture, cruel, inhuman or degrading punishment and treatment shall be prohibited.” Likewise, according to the European Court on Human Rights, slavery in the meaning of Article 4 ECHR requires exercise of a genuine right of ownership and “reduction of the status of the individual concerned to an ‘object’”.^23^ Furthermore, “exploitation through work”, this Court holds, qualifies as human trafficking as prohibited by Article 4 ECHR, since it “threatens the human dignity and fundamental freedoms of its victims”.^24^ These examples illustrate that, in international law, the wrongness of human exploitation relates, primarily, to its affront to human dignity. As a moral concept, human dignity has strong moral force, which has led to *absolute* prohibitions of practices disrespecting this fundamental value, including human exploitation.^25^

Note, finally, that some theorists, such as Wolff and Wertheimer, acknowledge different dimensions of “moral seriousness” of exploitation [29, 36]. As Jovanovic puts it, they tend to recognize different “levels of severity of exploitation”, which levels may correspond to, or justify, different forms of state intervention, ranging from no intervention at all to absolute prohibitions of specific exploitative behaviour [17, p. 692]. From a legal perspective, the UN Office on Drug and Crime considers this idea of a continuum of severe and less sever forms of exploitation “particularly useful”, because the “points on that continuum can be set with reference to the legal regime they fall within (and vice-versa)” [38,35, p. 14; 

For example, according to Jovanovic, within human rights law, the term ‘modern slavery’ encompasses practices that represent the severest forms of human exploitation, which States ought to criminalize in order to safeguard their citizens’ absolute right against those practices [17, p. 693]. Drawing on insights from the philosophical conceptualisation of exploitation, Jovanovic identifies three general, necessary and sufficient conditions that can help identifying such severest forms of human exploitation and distinguish them from other, closely related wrongs, such as extortion, abuse and fraud. These conditions are: (1) the abuse of vulnerability of the exploitee, (2) excessive (disproportionate) gain acquired through the actions of an exploitee, and (3) sustained action (the exploitative practice takes place over a period of time). These conditions, Jovanovic argues, “distinguish exploitation in the context of modern slavery from those practices that may well be considered exploitative within the general meaning of the term, but do not reach a level of severity necessary to trigger protection afforded by this ‘absolute’ right.” [17 p. 693]. They could serve as a normative tool to determine whether a given form of human exploitation should engage *absolute* protection from the prohibition of slavery, servitude, and human trafficking.

The next section explores whether the notion of exploitation as utilised in international law, may equally serve as a normative tool to assess whether different forms of mind interventions should engage absolute protection by the RFoT.

## The Exploitation of Mental Vulnerabilities and the RFoT: An Exploration

The twenty-first century has witnessed a marked increase in attention attributed to existing and emerging forms of human exploitation [15, 39]. Whereas different developments may explain this increase [39], from a legal perspective, Allain writes that it reflects a shift in emphasis from *victims* to *perpetrators* [15, p. 1]. That is, a shift in focus from victims’ *rights* against modern slavery, to criminal *prohibitions* of perpetrators’ actions involving modern slavery. The increased legal focus on exploitation, Allain notes, has also affected our understanding of this concept: “over time, society’s notion of exploitation has changed and the law has followed but also, in certain instances, the law has led. (…) What would have been deemed free labour one-hundred years ago or even fifty years ago, is seen now as having been exploitative. The standard by which we determine exploitation today is through law” [17, p. 2–3].

Thus far, the law has mainly guided our understanding of exploitation regarding practices that exploit human *bodies* (e.g., sexual exploitation, labour exploitation, organ removal). This section explores whether the exploitation of human *minds* should be a central concern of the law, too. More specifically, it explores whether the exploitation of persons’ mental vulnerabilities could be utilized as a normative tool to determine whether different mind interventions should qualify as the ‘impermissible alteration’ of thought and are therefore prohibited in absolute terms by the RFoT.

The exploitation language is regularly used in discussions on (the regulation of) practices that aim to influence people’s thoughts and decision-making by “exploiting mental vulnerabilities” [8, p. 12]. For example, Susser, Roessler and Nissenbaum describe how different digital means of influencing people’s beliefs, desires, emotions, habits and/or behavior sometimes operate through “exploiting their cognitive, emotional, or other decision-making vulnerabilities.” [13], p. 26]. For instance, as the OECD writes, “all dark patterns rely on cognitive mechanisms to influence the consumer (…) and many exploit specific biases or heuristics”, which has been characterised as strategies aiming to exploit automatic, intuitive decision-making with little cognitive effort (Kahneman’s System 1 thinking) [40, p. 9].

Considering the ethical, legal and regulatory challenges that emerge with the rise of the attention economy,^26^ the United Nations Economist Network has stressed that failure to protect individuals in this area “can lead to increasing authoritarian and surveillance regimes, with the ability to greatly influence collective human behavior by controlling the flow of information and exploiting human vulnerabilities” [41, p. 2]. Therefore, they argue for a regulatory framework that encourages the well-being and mutual benefit for all stakeholders, “rather than the current model that incentivize exploitation of individuals turning them into ‘objects of consumption’ instead of recognizing individuals’ human rights” [41, p. 5–6]. One principle that the Network think should guide policies in this area, is the principle of “freedom from exploitation”, which includes preventing “exploitation for *any reason*, at *all times*, and in *all spaces* whether physical or digital, even if those platforms or systems were not intended for those individuals as the primary audience” [41, p. 9, emphasis added].^27^

In the European context, a recent report produced under the mandate of the European Commission stresses that “Unfair business-to-consumer commercial practices like dark patterns and manipulative personalisation may (…) exploit consumer vulnerabilities”, and that the most effective remedy to protect consumers in this regard includes prohibitions of the most harmful practices [42, p. 6, 123]. In fact, some such practices, like dark patterns, are already prohibited under European law, exactly because of being exploitative. For example, Article 5.1(b) of the AI Act prohibits the use of AI systems that “exploit any of the vulnerabilities” inherent to certain individuals or groups of persons that could cause them significant harm. According to the European Commission, the notion of “vulnerabilities” in this context can be considered to “encompass a broad spectrum of categories, including cognitive, emotional, physical, and other forms of susceptibility that can affect the ability of an individual or a group of persons to make informed decisions or otherwise influence their behaviour” [43, at 102]. Meanwhile, although Article 5.1(b) refers to “any” vulnerability, the prohibition is limited to specific (groups of) persons defined by their age, disability, or socio-economic situations, who generally have more limited capacity to recognise or resist the AI influences or exploitative practices – for example, due to “reduced cognitive capacities” in children or older people – and are in need of enhanced protection [43, at 102–106].

Apart from mental influences that operate through digital techniques, *neurotechnologies* that enable control over another person’s mental states through direct brain intervention raise further concerns about the exploitation of the human mind. As Bublitz writes, neurotechnologies “provide a sometimes-unprecedented degree of control over another person, which can be misused and exploited. (…) More generally, as minds become more accessible, States and private actors may develop profound interests in new ways to exploit these novel powers to know and change how people think, feel, and behave” [24, p. 785]. In some cases, Bublitz suggests, mind interventions that exploit the person’s cognitive weaknesses may interfere with freedom of thought [8,44, p. 68]. Furthermore, considering ‘impermissible influences’ of thought in the meaning of the RFoT, Hertz writes that “[e]xploiting a person's need or weakness as well as an existing hierarchy are circumstances that may result in impermissible influence” [45, p. 12]., p. 21; 

Let’s assume, for the sake of argument, that some mind interventions indeed exploit the person’s cognitive weaknesses and interfere with mental states that qualify as thoughts in the meaning of the RFoT.^28^ How should the law in that case determine whether and when a specific exploitative intervention will infringe the absolute RFoT? Put differently: how should a legal doctrine on the RFoT distinguish between prohibited mind interventions and interventions that may well be considered exploitative within the general meaning of the term, but which do not reach the level of severity necessary to trigger absolute protection?^29^

One way of investigating this question is by drawing analogies between specific mind interventions on the one hand, and specific types of human exploitation that are absolutely prohibited by human rights on the other hand. For example, the absolute prohibition of slavery generally applies when one party has complete control or domination over another party. That is: in cases of *de jure* possession or the *de facto* exercise of powers over a person attaching to the right of ownership, reducing the exploited person to the status of an object; depriving them from making decisions themselves, e.g., by using, selling, or managing them [15,31, p. 219; 32, p. 287]. As Nowak/Schabas write in their commentary on the ICCPR, slavery forms the “extreme expression of the power human beings possess over their fellow human beings”, representing a direct attack on the essence of human personality and human dignity [21, p. 221]. Such “classic degradation” of persons to the mere property of another, they note, has given way to “highly sophisticated forms of personal dependence and economic exploitation” [21, p. 224]., p. 5; 

Perhaps, in analogy with the absolute prohibition of slavery, one may argue that the exploitation of mental vulnerabilities should be absolutely prohibited, too, if it disrespects self-ownership and dominates a person by controlling or managing their thoughts, feelings, or decision-making capacity, reducing the person to an object of nature, e.g., for the purpose of economic or political gain. Although the threshold of domination and disrespecting self-ownership in relation to mental states is high, some mind intervention may well qualify. One could think of some applications of neurotechnology (if ever available) that may be used to imprint thoughts, opinions, and/or feelings into another person’s mind, or to steer the person to make choices in a way tantamount to ‘possessing’ their decision-making capacity. Recognising that persons have (an interest in) self-ownership over their minds, Bublitz and Merkel have argued that:

It is the sinister side of neuroscientific techniques to bypass the first-person perspective and to treat the other as a physical object, reducing them to an object of nature, treating them according to natural laws and negating their control over their own minds. This heteronomous access to the inner realm of others may amount to mental programming, conditioning, brainwashing, and objectification. And this is what respect for human dignity forbids [44, p. 75].

Prohibiting such exploitative practices can be realized by applying the RFoT. That is, by qualifying such practices as the ‘impermissible alteration’ of thought. Whether this drawing of analogies between specific mind interventions and other types of human exploitation will indeed produce compelling reasons for granting absolute protection against certain types of mind interventions, is an open question that merits further research.

Another way to explore whether different types of mind interventions should trigger absolute protection by the RFoT – as a result of being exploitative – is by considering the necessary and sufficient conditions proposed by Jovanovic, which aim at assessing whether a given form of human exploitation should engage absolute protection within human rights law [17].^30^ In-depth examination of these conditions, their theoretical foundations and the possible implications for our understanding of the RFoT is beyond the scope of this paper. But let me provide some brief considerations regarding each condition, in the hope they may spark further debate.

### Abuse of the exploitee’s vulnerability

For an exploitative practice to qualify as modern slavery that should be prohibited in absolute terms, Jovanovic considers the abuse of some vulnerability or weakness as the minimum necessary condition [17, p. 694].^31^ In this regard she refers to an observation by the UN Office on Drug and Crime, that “in both politics and philosophy, ‘exploitation’, when attached to a person, is commonly understood as being linked to some weakness or vulnerability, which becomes the object of exploitation.” [35, p. 21].^32^ Such weaknesses or vulnerabilities can be of any kind, including physical, psychological or emotional [17, p. 695]. Importantly, Jovanovic stresses, it is the *abuse* of a person’s vulnerability that is a necessary condition – not the mere existence of the vulnerability per se. [p. 695]. Assessing abuse of a person’s vulnerability requires:

first, recognising specific traits as constituting vulnerability and, second, explaining the dynamics in which such vulnerability is played upon to ensure control over a victim. Hence, the notion of control (power) is inherent in this element and it is contingent upon a number of circumstances, which may not always stem from coercion [17, p. 696].

Considering the first requirement in relation to mind interventions, recall that some such interventions operate through targeting psychological or physical traits that qualify as “cognitive, emotional, or other decision-making vulnerabilities” [13, p. 26], or, as Bublitz puts it, “cognitive weaknesses” [8], such as biases, heuristics, or reduced cognitive capacities [40, 43]. Some means of influencing people’s minds intentionally play upon these vulnerabilities in order to gain control over the person’s attention, decision-making, thoughts, opinions, and/or behavior. Examples include addictive designs of online services, such as through gamification techniques, which, according to the European Parliament, target “psychosocial patterns playing on consumers’ psychological needs, vulnerabilities and desires (…) and playing into loss of self-control” [46, at J]. Furthermore, one could think of changing people’s thoughts or feelings through electrical modification of brain activity, abusing the person’s lack of control over the neural correlates of mental states.

### Excessive gain

Second, prohibited forms of exploitation in human rights law, Jovanovic argues, should involve excessive gain by the exploiter from the actions of the exploited person: exploitation “always implies the notion of excess – an unfair gain at the expense of an exploited person – distinguishing this type of wrong from others” [17, p. 697–698]. Whether such excessive gain by the exploiter – or “disproportionate burden” on the exploitee – exists in an individual case, is a question of facts, which needs to be determined on the basis of all circumstances of the individual case [17, p. 698–699]. Generally, the assessment of whether an exploitative practice involves excessive gain requires a balancing between the benefits to the exploiter and the losses of the exploitee [18, 36, 47].

Whether the employment of different types of mind interventions, in different contexts, plausibly put a disproportionate burden on the targeted person is therefore difficult to assess *in abstracto*. Moreover, it is a question that requires us to rethink and articulate how human rights should value the different interests at stake, including our interest in autonomous decision-making, regardless the outcomes and effects of those decisions. Although the condition of ‘excessive gain’ cannot be determined in the abstract, we can explore our intuitions by considering different types of cases, ranging from plausibly proportionate to plausibly disproportionate gain.

Consider, for example, a lucrative online service, such as a game or social media, which is designed to be highly addictive, so much that it may harm its users psychologically and financially – all in the name of profit. If such games are to be considered to abuse their users’ mental vulnerabilities, and are therefore exploitative, they seem to be a clear case of exploitation that involves excessive gain. Intuitively, making high profits at the expense of people’s mental health and economic well-being puts a disproportionate burden on the exploited party.^33^

Interestingly, the guidelines of the European Commission on the interpretation of Article 5.1(b) AI Act, which prohibits exploitative AI systems, explicitly refer to addictive games that exploit vulnerabilities, for example by the exploitation of children’s “limited ability to understand long term consequences, susceptibility to pressure, lack of self-control, and inclination towards instant gratification” [43, at 105]. As the Commission emphasises, such exploitative practices can have severe effects on children, like on their development and well-being, which may even extend into adulthood, including addictive behaviour, physical health problems, reduced cognitive capacities, concentration problems, and social difficulties. Article 5.1(b) prohibits such exploitative practices insofar as they “seriously harm” their users – but not when they (only) “bring benefits” [43, at 105]. For the prohibition of Article 5.1(b) to be applicable, an exploitative practice must distort the targeted person’s behavior in such a way that causes, or is reasonably likely to cause, “significant harm”, including physical, psychological, financial, and economic harms [43, at 115]. Making high profits by abusing cognitive vulnerabilities and seriously harming users by addictive designs may be a clear case of exploitation that involves excessive gain.

In other cases, meanwhile, the excessiveness of the gain generated by an (arguably) exploitative intervention may be less straightforward. Consider, for example, persuasive technologies that play upon persons’ cognitive weaknesses to steer them towards healthier lifestyle choices, such as quitting smoking. Although such influences may harm the subject in some way, for example, by taking away one’s joy in smoking a cigarette, the amount of the harm involved is arguably limited and could plausibly be considered proportionate to the benefits of these persuasive technologies, such as improving public health.

These examples illustrate the potential relevance of the distinction between harmful vs. harmless exploitation [18, 36]: if the exploitation of a person’s vulnerabilities causes them (serious) harm, the excessiveness of the gain acquired by the exploiter may be easier to assess. As Zwolinski, Ferguson, and Wertheimer write, one natural response to the question of what it means to benefit *unfairly* from another person, is to conceive of unfairness as benefiting *A* by harming *B* [30, at 2.1.3]. Some paradigmatic cases of exploitation, they write, easily fit into this idea, including slavery, which clearly constitutes an exploitative relationship that harms the slave for the benefit of the slaveholder.

Applying this reasoning to mind interventions could imply that – if they involve abuse of vulnerability (condition 1) – the presence of excessive gain and, therefore, the intervention’s unfairness, will become more plausible if they benefit the performer by (seriously) harming the targeted person. In those cases, the *unfairness* of the intervention may be considered an additional or aggravated wrong, in addition to the wrong of *harming* the victim.

### Sustained action

A final condition discussed by Jovanovic is that exploitation takes places – or is intended to take place – over a certain period of time. One-time abuse of a person’s vulnerability may be wrong, she argues, because it qualifies as fraud or abuse, “but exploitation in the context of modern slavery involves sustained activity” [17, p. 700]. Whereas some exploitative mind interventions typically operate over longer periods of time – such as dark patterns and addictive online designs – others may be of a more incidental nature, such as online advertisement targeting persons based on their occurrent emotional state,^34^ or the electrical stimulation of a person’s brain in the context of a short-term forensic treatment.

Whether the sustained nature of mind interventions would be a compelling condition for engaging absolute protection from the RFoT can be challenged. After all, incidental or short-term interferences can sometimes have significant effects on a person’s thoughts and thinking too. Some of them may even qualify as paradigmatic violations of the RFoT. One could think of hypothetical cases such as secretly administering a love-inducing drug to someone causing them to instantly fall in love with a specific person, or implanting or disrupting specific thoughts or thought processes by single-time electrical stimulation of a person’s brain. Although such interferences do not qualify as ‘sustained actions’, many would feel they will typically infringe the RFoT.^35^ Meanwhile, in some cases, the frequency and duration of a (relatively minor) mental influence might be relevant, for example, because sustained persistence of such influences may ultimately result in greater harm to the recipient and greater (potentially excessive) benefit to the performer. Examples may include constant and repeated online advertisements that target people’s occurrent emotional states, slowly inducing a strong desire to buy specific products, ultimately harming the person’s economic wellbeing. Perhaps, rather than a necessary condition to infringe the RFoT, the frequency and duration of an influence may serve as an aggravating factor [cf. 11, p. 332].

## Discussion: The Potential of Exploitation, Consent, and Challenges

The notion of exploitation is regularly used to describe a variety of modern forms of mental influence that raise ethical and legal concerns, including concerns about human rights. This paper aimed to explore whether the notion of exploitation could, apart from its descriptive use, also add to normative evaluations of mental influences under the RFoT. More specifically, it explored the relevance of ‘exploitation’ for the operationalisation of the freedom from ‘impermissible alteration of thought’.

Based on the analysis in this paper, I tentatively conclude that the notion of exploitation is, to some extent, a promising notion that can help identifying the boundaries of the RFoT’s absolute dimension. That is, by offering a principled basis to categorically outlaw severe forms of human exploitation that operate through the human mind by targeting mental vulnerabilities. Whereas the *prima facie* or *pro tanto* wrongness of ‘manipulation’ is often related to undermining *personal autonomy*, the wrongness of ‘exploitation’ is, in international law, primarily connected with contravening *human dignity*, being a central value underpinning human rights and often serving as a justification for grounding absolute legal prohibitions. In established human rights law, the gravest forms of human exploitation – slavery and servitude – are absolutely prohibited because of their inherently exploitative nature. Likewise, exploitation could serve as a normative tool to clarify what types of mental influences are – or should be – prohibited in absolute terms by the RFoT. These may include practices that exploit mental vulnerabilities to the extent that they compromise the person’s self-ownership over (certain) mental properties – dominating the person by controlling or managing their thoughts, feelings, or decision-making capacity, for example, to serve economic or political interests. Other candidate examples may include online services that exploit cognitive weakness and generate excessive gain at the expense of their users’ mental well-being.^36^

Employing the concept of exploitation for the operationalisation of the RFoT may have very concrete and practical implications for the regulation of mind interventions. One of them concerns the issue of consent. Since (severe) forms of human exploitation are considered to diminish human dignity, the law prohibits them in absolute terms and *irrespective of the victim’s consent* to the exploitative practice. For example, during the negotiations of the prohibition of slavery and servitude pursuant to Article 8 ICCPR, it was proposed to only prohibit “involuntary servitude”, expressing that the prohibition only dealt with “compulsory servitude and did not apply to normal contractual obligations between persons competent to enter into such obligations.” This proposal was rejected, because servitude in any form, whether involuntary or not, should be prohibited: “It should not be made possible for any person to contract himself into bondage.”^37^ Similar discussions had led to the removal of the word “involuntary” from earlier drafts of Article 4 UDHR.^38^ Unlike most other rights and freedoms, the freedom from severe exploitation cannot be waived simply by consenting to such practices.

In more recent legal instruments that aim to protect against human exploitation, the irrelevance of a person’s consent is made even more explicit. For example, Article 4 of the Council of Europe Convention on Action against Trafficking in Human Beings stresses that a victim’s consent to exploitative practices “shall be irrelevant” to the prohibition of human trafficking. A similar rule is enshrined in Article 3(b) of the Palermo Protocol. According to the UN Office on Drug and Crime, “considerations of ‘human dignity’ have been used to strengthen the position that one cannot consent (…) to one’s own exploitation – whatever form that exploitation takes” [48, p. 23]. As they note, a “competing – or at least balancing – liberal value (…) around consent has been ‘personal autonomy’ and the related value of respect for voluntary undertakings”, which “accepts the individual’s right to decide what is in his or her best interests and rejects attempts to invalidate such choices when these are rational and voluntary, even if they are patently unwise or likely to result in harm to the individual” [48, p. 23]. Whereas consent, when free and informed, is generally considered able to justify interferences with *personal autonomy*, it is regularly thought of as *not* being permitted to trump the fundamental value of *human dignity* and the protection of the most vulnerable people within society [48, p. 75].

In recent discussions over the regulation of emerging technologies that can access and alter private mental states, the issue of consent is a central concern too. Challenges include the voluntariness and transparency of consent in relation to opaque mental influences that operate below conscious awareness, as well as the implications of consent in relation to neurointerventions and the effective protection of vulnerable persons from human rights violations [9, 11, 12]. For example, in an attempt to clarify the meaning and scope of the freedom from ‘impermissible alteration’ of thought, the Special Rapporteur on freedom of religion and belief has suggested that a right holder’s consent can be a relevant factor when assessing whether a certain practice qualifies as “impermissible manipulation” of thought and therefore violates the RFoT [9]. Keese and Leiser, however, challenge this view. While accepting that consent “could theoretically be relevant”, they argue that consent should not always prevent the RFoT from being infringed, given the lack of opportunities for meaningful consent in current digital environments [12, p. 330]. Introducing the concept of exploitation to these discussions (instead of, or next to, manipulation), brings an alternative perspective to the fore: one in which consent may be *theoretically irrelevant*, regardless of the practical difficulties that arise across different societal contexts.

Utilizing exploitation to clarify the RFoT and to define the boundaries of its absolute dimension will, however, also entail challenges. For example, although exploitation is an international legal concept, it has been argued that it is still undefined in international law and lacks conceptual clearness [16, 17]. This, it might be argued, speaks against the use of exploitation to operationalise the RFoT, as introducing yet another indeterminate concept – alongside an already substantial number of ambiguous notions (such as thought, manipulation, coercion, modulation, consent, absolute protection) – is unlikely to increase conceptual clarity and may even exacerbate existing ambiguities. At the same time, exploitation may also mitigate some of the existing unclarities, for example, by replacing the notion of manipulation and by identifying mental influences that plausibly deserve absolute protection.

Furthermore, legal scholars such as Jovanovic are now aiming to develop a clearer understanding of exploitation by identifying its necessary conditions. Whereas these endeavours contribute to getting a clearer understanding of exploitation as an international legal concept, they also raise novel questions. For example, whereas Jovanovic’s first condition – abuse of a person’s vulnerability – is more or less straightforward, the second condition of excessive gain turns out to be much more complex and may raise doubts about the suitability of exploitation for the operationalisation of the RFoT. First, assessing whether a specific practice involves *unfair gain* at the expense of the exploited person entails a *balancing* of the benefits and losses of each party in view of all relevant circumstances of an individual case. At first sight, such a balancing exercise appears to sit uneasily with the idea that the RFoT is an absolute human right.^39^ Furthermore, different theories exist on what is fair and what is unfair, and how (un)fairness should be determined – something that may also depend on political and cultural differences. How should the law deal with this? And do we actually need to settle all these issues beforehand, or can some elements of the notion of exploitation be left open for interpretation by international and domestic courts, slowly developing a standard through which the law will determine whether different types of exploitative mind interventions would qualify as excessive and unfair?^40^

Another question that arises regarding the potential use of exploitation for the operationalisation of the RFoT, is *how* it should be used. Should exploitation become a necessary and/or sufficient condition for mind interventions to infringe the freedom from impermissible alternation of thought? Or should it just be one relevant factor together with other factors relevant in determining whether absolute protection from the RFoT is available?^41^ Addressing these questions is beyond the scope of this paper, but I hope that they will spur further research into the potential of exploitation for the operationalisation of the RFoT.

## Conclusion

One central principle often derived from the RFoT, is that persons’ inner thoughts shall not be impermissibly altered. Since a clear definition of ‘impermissible alteration’ of thought is lacking, the meaning and scope of this principle are largely uncertain. Scholars are now exploring how to operationalise the notion of ‘impermissible alteration’ of thought. For this, some have appealed to the concept of ‘manipulation’, proposing that mind interventions plausibly infringe the RFoT if they are manipulative. But appealing to manipulation in this context is challenging for several reasons. Most importantly, manipulation is usually considered to be a *prima facie* and/or *pro tanto* wrong, which can be justified sometimes, while the RFoT is generally considered to be an *absolute* right, not allowing for any justifications whatsoever.

This paper explored the normative relevance of the distinct notion of *exploitation*, which is, unlike manipulation, an international legal concept that underpins different absolute prohibitions in human rights law. To some extent, exploitation appears a promising concept to identify the boundaries of the RFoT’s absolute dimension. Its strong moral force can contribute to justifying categorical prohibitions of some types of mind interventions. Grounding such absolute protection in the notion of exploitation can furthermore have very specific implications for the regulation of certain mental interventions, such as the legal irrelevance of the person’s consent to exploitative interventions. Meanwhile, this paper showed that introducing exploitation to debates on the RFoT also brings new challenges, such as regarding the concept’s definition in international law, the meaning of ‘excessive gain’, and its suitably to define ‘absolute’ rights due to the balancing of gains and losses. The analysis in this paper may serve as a starting point for further research on the utility of exploitation for the legal understanding and operationalisation of the RFoT.

## Footnotes

1 When referring to ‘mind interventions’, this paper means actions undertaken by an agent to influence the. mind of an individual, for example, by changing a person’s thoughts, desires, or intentions. Cf. [2].

2 For critical reflection on this issue, see [23].

3 In human rights law, justifiable wrongs are typically protected against by relative rights that include specific requirements to justify rights infringements, such as the right to personal integrity inherent in Article 8 ECHR.

4 Note that in human rights law, the absolute nature of the prohibition of human trafficking is not (yet) fully clear. For example, although the ECtHR has stressed that human trafficking is covered by the prohibition of slavery, servitude and forced labour pursuant to Article 4 ECHR, it has not clarified whether the prohibition is absolute (like the prohibition of slavery and servitude) or nearly-absolute (like the prohibition of forced labour), see ECtHR 7 January 2010, 25,965/04 *(Rantsev v. Cyprus and Russia*), par. 282.

5 For discussion see [23].

6 In proposing these factors, Keese and Leiser build upon four potentially relevant factors to assess whether certain practices impermissibly manipulate people’s thoughts as mentioned in the 2021 report from the Special Rapporteur on freedom of religion or belief [9, par. 36].

7 Which may apply, according to Nowak/Schabas, to a range of different means that interfere with or influence the mind, including “through indoctrination, ‘brainwashing’, influencing of the conscious or subconscious mind with psychoactive drugs or other means of manipulation.” Meanwhile, they stress that the difference between impermissible and permissible influencing is not easily made. In any event, influencing is “impermissible when it is performed by way of coercion, threat or some other prohibited means against the will of the person concerned or without at least his or her implicit approval” [[21], at 503].

8 See also [[49], p. 18]. Or, as the Special Rapporteur puts it, to the freedom from impermissible *alteration* of thought [[9], p. 28].

9 Which, however, need not always be the case [50, 51]. Moreover, autonomy undermining practises are usually considered to be justifiable on balance.

10 Which view has been endorsed by Alegre and McCarthy-Jones [6, 7]. But see also McCarthy-Jones in later work, exploring arguments against an *absolute* RFoT [[52], p. 263].

11 A question that has led to much discussion in the philosophical literature, also in relation to digital technologies. See, e.g., [53].

12 Cf. [12, 54].

13 ECtHR (GC) 26 April 2016, 62,649/10 (*İzzettin Doğan and others v. Turkey*), § 68.

14 In the context of the RFoT, *exclusively* focusing on actions by the performer seems counterintuitive, that is, if one assumes that the right essentially aims to protect rational capacity or mental autonomy of the *victim*. I thank Kelly Dhru for raising this point.

15 See, e.g., [18, 36].

16 Elsewhere, Wood illustrates this further, if he writes: “When it is said that the surrogacy exploits the mother, however, the idea is not *only* that surrogacy exploits her labor (…); the point is rather that it exploits her ability to carry and give birth to a child, which is taken to be such an intimate part of what she is that it could not be exploited without also exploiting *her*” [[37], p. 146, original emphasis].

17 See also Article 16 of the Convention on the Rights of Persons with Disabilities.

18 Cf. Article 4 of the Council of Europe Convention on Action against Trafficking in Human Beings.

19 Concurring opinion, Judge Pinto de Albuquerque, joined by Judge Tsotsoria, ECtHR 17 January 2017, 58,216/12 (J. and others v. Austria), par. 38, 40; CCPR General Comment No. 29: Article 4: Derogations during a State of Emergency, par. 11–12.

20 As he writes: “There is always a presumption in favor of fairness and against unfairness, but there is no necessary correspondence between on-balance justification and fairness. They are quite distinct notions, and, in this imperfect world, only imperfectly linked [[18], p. 200].

21 See also [16], p. 300, stating that “the core idea of treating someone as the instrument of one’s own ends is invariably there”. Note that the meaning and use of human dignity in human rights law is subject to considerable debate. In this paper, human dignity is understood as a fundamental guiding principle of human rights, affirming the equal inherent worth of all human beings as autonomous human beings able to pursue a meaningful life in relation with others.

22 E/CN.4/AC.1/3/Add.1, p. 59; E/CN.4/21, p. 11.

23 ECtHR 7 January 2010, 25,965/04 *(Rantsev v. Cyprus and Russia*), par. 276.

24 ECtHR 30 March 2017, 21,884/15 (*Chowdury and Others v. Greece*), par. 93.

25 See, for example, the absolute prohibition of slavery and servitude and the absolute prohibition of torture, inhuman and degrading treatment.

26 I.e., an approach to the management of information that treats human attention as a scarce commodity and applies economic theory to solve information management problems. In this regard, technologies have been increasingly employed to capture persons’ attention supported by the collection and analysis of personal data, which has proved to be a very profitable business model [41].

27 Additionally, “there should be special considerations and protections for children and the elderly in all attention-based systems.”

28 For a discussion on how to define ‘thought’, see [54–56].

29 Such mind interventions may still be protected against by relative human rights, including the right to personal integrity. The same applies to mind interventions that are not exploitative at all but are (*prima facie*) impermissible for other reasons.

30 Which Jovanovic considers in view of modern slavery.

31 In human rights law, the labeling approach to vulnerability appears dominant, qualifying certain groups of people more vulnerable to harm than others, which justifies enhanced human rights protection [57, 58]. For critique on the labeling approach see, e.g., the work of Fineman [59].

32 See also p. 4: “a number of countries have integrated abuse of vulnerability into their understanding of exploitation’.

33 Cf. [[47], p. 145].

34 Such advertisement strategies could of course also be applied for longer periods of time.

35 Cf. the practice of human trafficking for the purpose of organ removal, which is considered a one-time practice but nevertheless qualifies as human exploitation that is strictly prohibited in international law, for example by Article 4 ECHR.

36 Some such practices may also qualify as manipulation on some accounts and would, according to some authors, therefore engage protection from the RFoT, see, e.g., [12]. One of the relevant differences, however, is that unlike manipulation (on many accounts), exploitation need not be hidden or covert. And unlike exploitation, manipulation is not typically linked to unfairness and undermining human dignity.

37 A/2929, p. 92.

38 Earlier drafts of Article 4 UDHR only entailed an absolute prohibition of “involuntary servitude”. But the Colombian representative was unsatisfied with this, as “servitude of any kind was inadmissible” [[60], p. 2051]. The Venezuelan representative agreed, highlighting that the UDHR “should cover all forms of slavery and not only the most obvious ones. In modern times there was a new form of slavery brought about by economic conditions” [[60], p. 2306]. The Ukrainian representative added that including the word “involuntary” may “provide an escape clause, as slave owners would be able to evade the provisions of the declaration by saying that their slaves had entered into servitude voluntarily” [[60], p. 2308]. The word “involuntary” was ultimately removed [[60], p 2311].

39 Cf. discussions over the ‘absolute’ nature of the prohibition of ill-treatment in relation to excessive vs. non-excessive force [61]. See also [62].

40 Cf. the evolution of ‘exploitation’ in the context of modern slavery [26, 39], and recall Allain’s remark that “over time, society’s notion of exploitation has changed and the law has followed but also, in certain instances, the law has led. (…) The standard by which we determine exploitation today is through law” [13, p. 2–3].

41 For example, in the context of a *de minimis* threshold for the RFoT’s applicability [63].

## References

[1] O’Callaghan, P., and B. Shiner. 2025. *The Cambridge handbook for freedom of thought*. Cambridge: CUP.

[2] Istace, T. 2025. Exploring the essence of the freedom of thought. 2025. *Neuroethics* 18:10.

[3] Teo, S. A. 2024. How to think about freedom of thought (and opinion) in the age of AI. *Computer Law & Security Review* 53:105969.

[4] Ligthart, S., et al. 2022. Rethinking the right to freedom of thought: A multidisciplinary analysis’. *Human Rights Law Review* 2022:1–14.

[5] Blitz, M. J., and J. C. Bublitz. 2021. *The law and ethics of freedom of thought, Volume 1*. Cham: Palgrave Macmillan.

[6] Alegre, S. 2023. *Freedom to think*. London: Atlantic Books.

[7] McCarthy-Jones, S. 2019. The autonomous mind: The right to freedom of thought in the twenty-first century’. *Frontiers in Artificial Intelligence* 2:1–17. 33733108 10.3389/frai.2019.00019 PMC7861318

[8] Bublitz, J. C. 2014. Freedom of thought in the age of neuroscience. *Archiv Für Rechts- Und Sozialphilosophie* 100:1–25.

[9] Special Rapporteur on Freedom of Religion or Belief. 2021. Freedom of thought. A/76/380.

[10] Vermeulen, B. and Roosmalen, M. (2018). Freedom of thought, conscience and religion. In *Theory and Practice of the European Convention on Human Rights*, Van Dijk, P., et al. (eds.), Cambridge: Intersentia 2018.

[11] Advisory Committee, UNHRC. 2024. Impact, opportunities and challenges of neurotechnology with regard to the promotion and protection of all human rights, 8 August 2024, A/HRC/AC/31/CRP.1.

[12] Keese, N. and Leiser, M. 2025. Online manipulation as a potential interference with the right to freedom of thought. In *The Cambridge Handbook for Freedom of Thought*, eds, O’Callaghan, P. and Shiner, B. Cambridge: CUP.

[13] Susser, D., B. Roessler, and H. Nissenbaum. 2019. Online manipulation: Hidden influences in a digital world. *Georgetown Law Technology Review* 4:1–45.

[14] Sunstein, C. 2022. Manipulation as theft. *Journal Of European Public Policy* 29:1959–1969.

[15] Allain, J. 2013. *Slavery in international law: Of human exploitation and trafficking*. Leiden: Nijhoff.

[16] Marks, S. 2008. Exploitation as an international legal concept. In *International Law on the Left,* Marks, S. (ed). Cambridge: CUP.

[17] Jovanovic, M. 2020. The essence of slavery: Exploitation in human rights law. *Human Rights Law Review* 20:674–703.

[18] Feinberg, J. 1990. *The moral limits of the criminal law volume 4: Harmless wrongdoing*. Oxford: OUP.

[19] Noggle, R. The ethics of manipulation. *The Stanford Encyclopedia of Philosophy *(Summer 2022 Edition), Edward N. Zalta (ed.), Summer 2022.

[20] Alegre, S. 2017. Rethinking freedom of thought for the 21st century. *E.H.R.L.R.* 3: 221–233.

[21] Nowak, M./Schabas, W. (2019). *U.N. International Covenant on Civil and Political Rights: Nowak’s CCPR Commentary*, 3^rd^ revised edition, Kehl: N.P. Engel.

[22] Scott, R.M. 2021. A Short-Term Option for Addressing Misinformation during Public Health Emergencies: Online Nudging and the Human Right to Freedom of Thought. *OpinioJuris* (online).

[23] Ligthart, S. 2025. Reconsidering the absolute nature of the right to freedom of thought. *Human Rights Law Review* 25:1–27.

[24] Bublitz, J. C. 2024. Neurotechnologies and human rights: Restating and reaffirming the multi-layered protection of the person. *The International Journal of Human Rights* 28:782–807.

[25] Ligthart, S. and Van de Pol, N. 2025. Freedom of thought: absolute protection of mental privacy and mental integrity? In *The Cambridge Handbook for Freedom of Thought*, eds. O’Callaghan, P. and Shiner, B. Cambridge: CUP.

[26] Allain, J. 2015. *The Law and Slavery: Prohibiting Human Exploitation*. Leiden: Brill Nijhof.

[27] Allain, J. 2008. The Slavery Conventions: The Travaux Préparatoires of the 1926 League of Nations Convention and the 1956 United Nations Convention. Leiden: Brill Nijhof.

[28] Wood., W. 2014. Coercion, manipulation, exploitation. *In Manipulation: Theory and Practice,* Coons, C., and Weber, M. (eds.). Oxford: OUP.

[29] Wolff, J. 1999. Marx and exploitation. *Journal of Ethics* 3:105–120.

[30] Zwolinski, M., Ferguson, B. and Wertheimer, A. (2022). Exploitation, In *The Stanford Encyclopedia of Philosophy *(Winter 2022 Edition), Edward N. Zalta and Uri Nodelman (eds.).

[31] Taylor, P. M. 2020. *A commentary on the international covenant on civil and political rights*. Cambridge: CUP.

[32] Harris, D.J., et al. 2023. *Harris, O’Boyle, and Warbrick: Law of the European Convention on Human Rights*. New York: OUP.

[33] Office of the prosecutor 2024, *Policy on slavery crimes*.

[34] Sellers, P. V., and J. G. Kestenbaum. 2020. Missing in action: The international crime of the slave trade. *Journal of International Criminal Justice* 15:517–542.

[35] UNODC. 2015. *The concept of ‘exploitation’ in the trafficking in persons protocol*. Vienna: United Nations.

[36] Wertheimer, A. 1996. *Exploitation*. Princeton: Princeton University Press.

[37] Wood, A. 1995. Exploitation. *Social Philosophy and Policy* 12:136–158.

[38] UNODC. 2018. *The International Legal Definition of Trafficking in Persons: Consolidation of research findings and reflection on issues raised*. Vienna: United Nations.

[39] Chuang, J. A. 2014. Exploitation creep and the unmaking of human trafficking law. *The American Journal of International Law* 108:609–649.

[40] OECD. 2022. Dark commercial patterns. DSTI/CP(2021)12/FINAL.

[41] United Nations Economist Network. 2023. New economics for sustainable development: Attention economy. Policy Brief.

[42] Lupiáñez-Villanueva, F. et al. 2022. Behavioural study on unfair commercial practices in the digital environment: dark patterns and manipulative personalization. Brussles: European Commission.

[43] EU Commission. 2025. Commission guidelines on prohibited artificial intelligence practices. C(2025) 884 Final.

[44] Bublitz, J. C., and R. Merkel. 2014. Crimes against minds: On mental manipulations, harms and a human right to mental self-determination. *Criminal Law and Philosophy* 8:51–77.

[45] Hertz, N. 2025. The human right to freedom of thought—operationalising a disputed right in the context of neurotechnologies. *Human Rights Law Review* 25:1–22.

[46] European Parliament. 2023. Report on addictive design of online services and consumer protection in the EU single market. A9–0340/2023.

[47] Mayer, R. 2007. What is wrong with exploitation? *Journal Of Applied Philosophy* 24:137–150.

[48] UNODC. 2014. *The role of ‘consent’ in the trafficking in persons protocol*. Vienna: United Nations.

[49] Murdoch, J. 2012. Protecting the right to freedom of thought, conscience and religion under the European Convention on Human Rights. Strasbourg: Council of Europe.

[50] Blumenthal-Barby, J. S. 2012. Between reason and coercion: Ethically permissible influence in health care and health policy contexts. *Kennedy Institute of Ethics Journal* 22:345–366. 23420941

[51] Buss, S. 2005. Valuing autonomy and respecting persons: Manipulation, seduction, and the basis of moral constraints. *Ethics* 115:195–325.

[52] McCarthy-Jones, S. 2023. *Freethinking*. London: Oneworld Publications.

[53] Jongepier, F., and M. Klenk. 2022. *The philosophy of online manipulation*. New York: Routledge.

[54] Bublitz, J.C. 2025. What is Thought? Interpreting and Constructing Article 18 ICCPR in Light of the Vienna Convention. In *The Law and Ethics of Freedom of Thought Vol. 2: Cognitive Liberty and Privacy*, eds. Blitz, M. and J.C. Bublitz, J.C. Cham: Palgrave McMillan 2025 (in press).

[55] Ligthart, S. Mental privacy as part of the human right to freedom of thought?, in M. Blitz and J.C. Bublitz (eds.), *The Law and Ethics of Freedom of Thought Vol. 2: Cognitive Liberty and Privacy*, Palgrave McMillan (in press).

[56] Ligthart, S. What is thought, or what should it be? – Reply to Bublitz, in M. Blitz and J.C. Bublitz (eds.), *The Law and Ethics of Freedom of Thought Vol. 2: Cognitive Liberty and Privacy*, Palgrave McMillan (in press).

[57] Morawa, A. H. E. 2023. Vulnerability as a concept of international human rights law. *Journal of International Relations and Development* 6:139–155.

[58] Peroni, L., and A. Timmer. 2013. Vulnerable groups: The promise of an emerging concept in European Human Rights Convention law. *International Journal of Constitutional Law* 11 (4): 1056–1108.

[59] Fineman, M. A. 2008. The vulnerable subject: Anchoring equality in the human condition. *Yale Journal of Law & Feminism* 20 (1): 1–23.

[60] Schabas, W.A. 13. *The universal declaration of human rights: The Travaux Préparatoires*, New York: CUP.

[61] Nowak, M. 2005. Challenges to the absolute nature of the prohibition of torture and ill-treatment. *Netherlands Quaterly of Human Rights* 23:674–688.

[62] Borowski, M. 2013. Absolute rights and proportionality. *German Yearbook of International Law* 56:385–423.

[63] Shiner, B. Threshold to operationalize the right. Talk at the Workshop on *The right to freedom of thought and neurotechnology*, Oxford, 22 January 2025.

